# Inhibition of Butyrylcholinesterase and Human Monoamine Oxidase-B by the Coumarin Glycyrol and Liquiritigenin Isolated from *Glycyrrhiza uralensis*

**DOI:** 10.3390/molecules25173896

**Published:** 2020-08-26

**Authors:** Geum Seok Jeong, Myung-Gyun Kang, Joon Yeop Lee, Sang Ryong Lee, Daeui Park, MyoungLae Cho, Hoon Kim

**Affiliations:** 1 Department of Pharmacy, and Research Institute of Life Pharmaceutical Sciences, Sunchon National University, Suncheon 57922, Korea; fever41@naver.com (G.S.J.); wholsr@naver.com (S.R.L.); 2 Department of Predictive Toxicology, Korea Institute of Toxicology, Daejeon 34114, Korea; myung-gyun.kang@kitox.re.kr (M.-G.K.); daeui.park@kitox.re.kr (D.P.); 3 National Institute for Korean Medicine Development, Gyeongsan 38540, Korea; chool9090@nikom.or.kr (J.Y.L.); meanglae@nikom.or.kr (M.C.)

**Keywords:** *Glycyrrhiza uralensis*, glycyrol, liquiritigenin, cholinesterases, human monoamine oxidases, kinetics, docking simulation

## Abstract

Eight compounds were isolated from the roots of *Glycyrrhiza uralensis* and tested for cholinesterase (ChE) and monoamine oxidase (MAO) inhibitory activities. The coumarin glycyrol (GC) effectively inhibited butyrylcholinesterase (BChE) and acetylcholinesterase (AChE) with IC_50_ values of 7.22 and 14.77 µM, respectively, and also moderately inhibited MAO-B (29.48 µM). Six of the other seven compounds only weakly inhibited AChE and BChE, whereas liquiritin apioside moderately inhibited AChE (IC_50_ = 36.68 µM). Liquiritigenin (LG) potently inhibited MAO-B (IC_50_ = 0.098 µM) and MAO-A (IC_50_ = 0.27 µM), and liquiritin, a glycoside of LG, weakly inhibited MAO-B (>40 µM). GC was a reversible, noncompetitive inhibitor of BChE with a K_i_ value of 4.47 µM, and LG was a reversible competitive inhibitor of MAO-B with a K_i_ value of 0.024 µM. Docking simulations showed that the binding affinity of GC for BChE (−7.8 kcal/mol) was greater than its affinity for AChE (−7.1 kcal/mol), and suggested that GC interacted with BChE at Thr284 and Val288 by hydrogen bonds (distances: 2.42 and 1.92 Å, respectively) beyond the ligand binding site of BChE, but that GC did not form hydrogen bond with AChE. The binding affinity of LG for MAO-B (−8.8 kcal/mol) was greater than its affinity for MAO-A (−7.9 kcal/mol). These findings suggest GC and LG should be considered promising compounds for the treatment of Alzheimer’s disease with multi-targeting activities.

## 1. Introduction

Acetylcholinesterase (AChE, EC 3.1.1.7) catalyzes the breakdown of acetylcholine (ACh), a neurotransmitter found in synapses of the cerebral cortex [1]. AChE inhibitors reduce AChE activity and maintain or increase ACh levels, which are typically deficient in Alzheimer’s disease (AD), and thus, enhance cholinergic transmission in brain [2,3]. AD is a chronic, devastating manifestation of neuronal dysfunction and is characterized by progressive mental failure, disordered cognitive functions, and speech impairment. Various cholinesterase inhibitors (e.g., tacrine, donepezil, galantamine, and rivastigmine), immunotherapies, antisense oligonucleotides, phyto-pharmaceuticals, and nutraceuticals are being used to treat AD [4].

Butyrylcholinesterase (BChE) breaks down butyrylcholine (BCh), and BChE levels are significantly elevated in the AD brain [5,6]. Interestingly, AChE and BChE, which are both related to AD and act independently, are viewed as diagnostic markers and as potential targets for drug development [7].

On the other hand, monoamine oxidases (MAO, EC 1.4.3.4) catalyze the oxidative deamination of monoamine neurotransmitters and are present as two MAO isoforms, that is, MAO-A and MAO-B, in the outer mitochondrial membranes of all tissues [8]. MAO-A is primarily targeted to treat depression and anxiety, whereas MAO-B is targeted to treat AD and Parkinson’s disease (PD) [9]. In addition, MAOs are critically related to amyloid plaque formation in AD, and MAO-B is co-expressed at high levels in the AD brain with γ-secretase [10].

Due to the complex etiology of AD, multi-targeting therapeutic agents have been devised to inhibit MAOs and AChE, and thus, elevate monoamine and choline ester levels [11]. Recently, multi-targeting agents such as homoisoflavonoid derivatives [12], donepezil-butylated hydroxytoluene hybrids [13], coumarin-dithiocarbamate hybrids [14], alcohol-bearing dual inhibitors [15], and chalcone oxime ethers [16] have been reported to target MAO-B and AChE. Dual function inhibitors of AChE and BChE have been studied using in silico approaches, such as pharmacophore-based virtual screening and molecular docking [17]. In addition, compounds targeting for MAO-A, MAO-B, AChE, and BChE like TV 3326 [18] and 1,2,3,4-tetrahydrochromeno[3,2-c]pyridin-10-one derivatives [19] have also been described.

Glycyrol (GC), a coumarin derivative, has been reported to have anticancer [20,21,22], anti-fungal [23], anti-bacterial [24], anti-viral [25,26], anti-inflammatory [27,28,29], and immunosuppressive activities [30]. However, the ChE inhibitory activity of GC has not been reported to date. Liquiritigenin (LG), a flavonoid, is also known to have many biological activities including MAO inhibitory activity [31,32]. However, these MAO studies were conducted using fractions of rat liver mitochondrial MAO and rat brain MAO-A and MAO-B, respectively.

In the present study, eight compounds were isolated from *Glycyrrhiza uralensis* (also known as Chinese licorice) and investigated for their inhibitory activities against AChE, BChE, and human MAO-A and MAO-B. The two most potent compounds (GC and LG) were subjected to kinetic analysis and their affinities for the enzymes were investigated using molecular docking simulations.

## 2. Results

### 2.1. Isolation and Identification of Compounds

**Figure 1:**
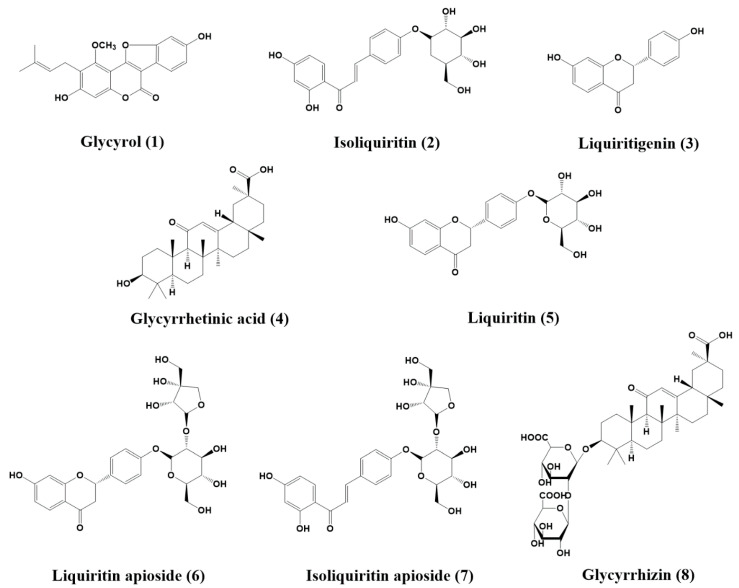
Chemical structures of eight compounds isolated from the roots of *Glycyrrhiza uralensis.*

Eight natural products were isolated from *Glycyrrhiza uralensis* and identified by comparing NMR data (Supplementary Information) with literature values: GC (**1**) [33], isoliquiritin (**2**) [33], LG (**3**) [33], glycyrrhetinic acid (**4**) [34], liquiritin (**5**) [33], liquiritin apioside (**6**) [35], isoliquiritin apioside (**7**) [36], and glycyrrhizin (**8**) [37]. The structures of the eight compounds are detailed in Figure 1.

### 2.2. Analysis of Inhibitory Activities

The AChE, BChE, MAO-A, and MAO-B inhibitory activities of the eight compounds were investigated at a concentration of 10 µM. Most of the eight inhibited AChE, BChE, MAO-A, and MAO-B by less than 50%. However, GC and LG potently inhibited AChE and BChE, and MAO-A and MAO-B, respectively (Table 1). GC inhibited BChE and AChE with IC_50_ values of 7.22 and 14.77 µM, respectively, with a selectivity index (SI) of 2.0 for BChE with respect to AChE, and also moderately inhibited MAO-B (29.48 µM). Other compounds showed weak inhibitory activities against AChE or BChE, except liquiritin apioside, which moderately inhibited AChE (IC_50_ = 36.68 µM). LG potently inhibited MAO-B (IC_50_ = 0.098 µM) and MAO-A (IC_50_ = 0.27 µM). The SI value of LG for MAO-B with respect to MAO-A was 2.8 (Table 1). Liquiritin, a LG glycoside, weakly inhibited MAO-A and MAO-B (>40 µM). Thus, GC and LG were found to be effective inhibitors of BChE and MAO-B, respectively.

### 2.3. Analysis of the Reversibilities of BChE and MAO-B Inhibitions

**Figure 2:**
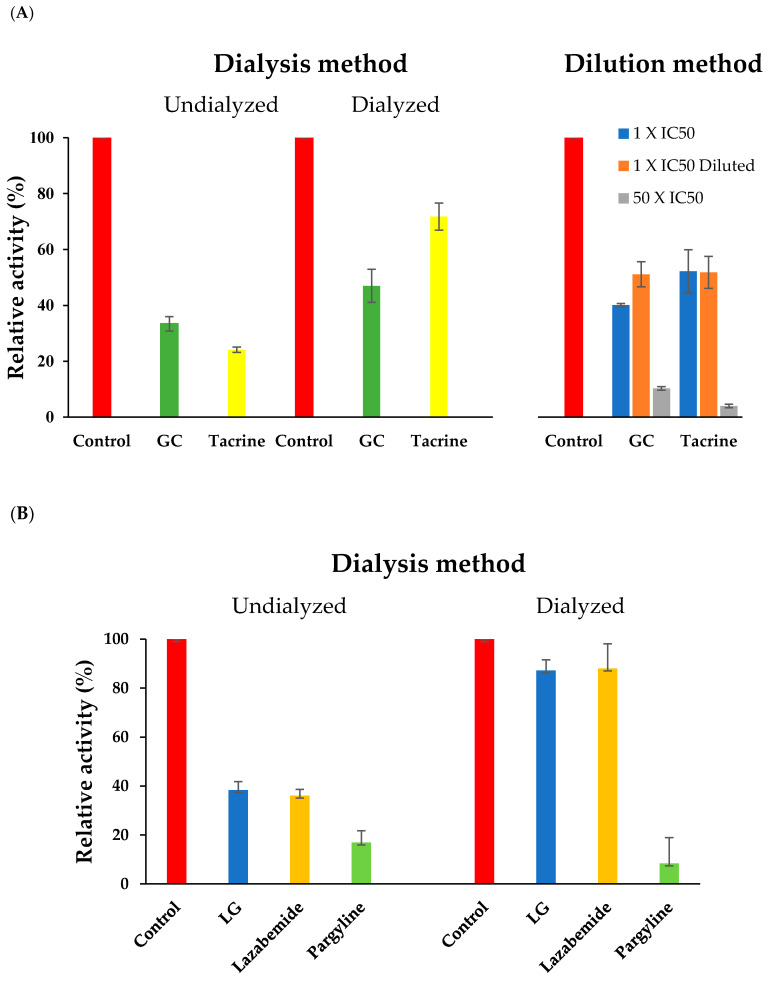
Recoveries of BChE inhibition by GC (**A**) and MAO-B inhibition by LG (**B**). The concentrations of the inhibitors used were: GC, 14.0 µM; LG, 0.2 µM; tacrine, 0.03 µM; lazabemide, 0.12 µM; and pargyline, 0.06 µM. In the dilution experiments, we measured residual activities of BChE at an inhibitor concentration of 1 × IC_50_, at a concentration of 50 × IC_50_ and then diluted to a concentration of 1 × IC_50_ after preincubation, at an inhibitor concentration of 50 × IC_50_. Results are the averages of duplicate or triplicate (GC dialysis) experiments. Tacrine was used as a reversible reference BChE inhibitor. Lazabemide and pargyline were used as reversible and irreversible reference MAO-B inhibitors, respectively.

Reversibilities of BChE inhibition by GC and of MAO-B inhibition by LG were investigated by dialysis and dilution methods. Residual BChE activity after GC inhibition recovered partially from 34.6% (undialyzed activity; A_U_) to 58.4% (dialyzed activity; A_D_) by dialysis, whereas inhibition by tacrine (a known reversible inhibitor) significantly recovered from 10.3% to 74.1% (Figure 2A). We also confirmed reversibility using the dilution method by measuring and comparing residual BChE activities of a sample preincubated with GC at a concentration of 50 × IC_50_ and then diluted to a concentration of 1 × IC_50_ with a control sample treated at a GC concentration of 1 × IC_50_. We found that residual activities were similar before and after dilution (51.1% and 40.1%, respectively), and that the activity of the sample at a concentration of 50 × IC_50_ was 10.3% (Figure 2A). These results suggested that GC is a reversible inhibitor of BChE, because if it acted as an irreversible inhibitor, activity would have been reduced by dilution. On the other hand, the relative residual activity of MAO-B after LG inhibition recovered from 38.4% (A_U_) to 87.2% (A_D_) by dialysis, which was similar to activity recovery observed for the reversible MAO-B inhibitor lazabemide (from 36.1% to 88.0%). On the other hand, values for the irreversible inhibitor pargyline were 17.0% and 8.4%, respectively (Figure 2B). These results showed that GC and LG reversibly inhibited BChE and MAO-B, respectively.

### 2.4. Analysis of Inhibitory Patterns

**Figure 3:**
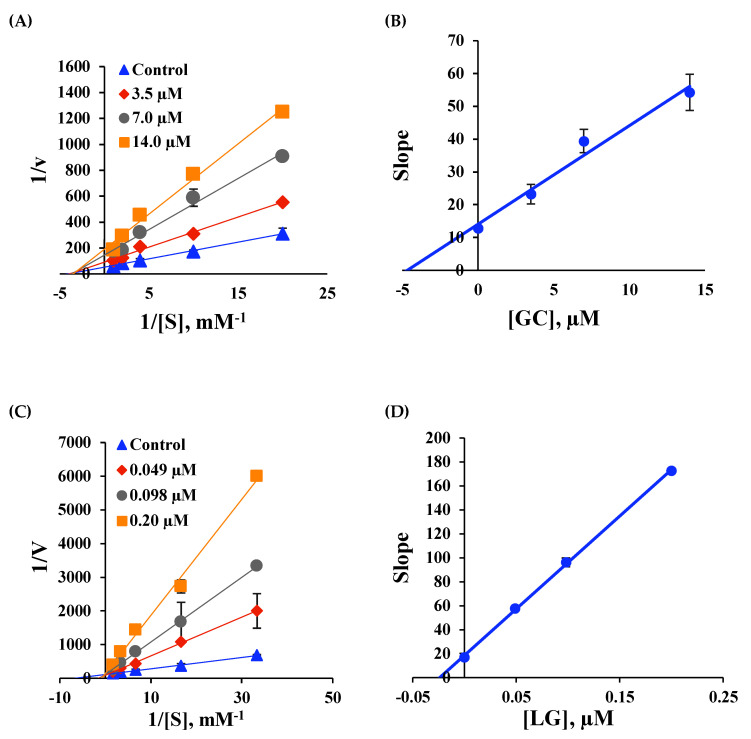
Lineweaver-Burk plots of inhibitions of BChE by GC (**A**) and of MAO-B by LG (**C**), and their respective secondary plots (**B**,**D**) of slopes of Lineweaver-Burk plots versus inhibitor concentrations. Five different substrate concentrations were used; 0.05, 0.1, 0.25, 0.5 or 1.0 mM for BChE, and 0.03, 0.06, 0.15, 0.3, or 0.6 mM for MAO-B. Inhibition studies were carried out at three inhibitor concentrations, that is, at 0.5×, 1.0×, and 2.0× of the IC_50_ values of GC and LG. The errors were determined by duplicate experiments.

Modes of BChE inhibition by GC and of MAO-B inhibition by LG were investigated by analyzing Lineweaver–Burk plots. Plots of BChE inhibition by GC were linear and intersected the *x*-axis (Figure 3A). Secondary plots of the slopes of Lineweaver–Burk plots against inhibitor concentration showed the K_i_ value of GC for BChE inhibition was 4.47 ± 0.29 µM (Figure 3B).

These results indicate GC acted as a noncompetitive inhibitor of BChE and bound to a site other than the understood substrate binding site of BChE. On the other hand, plots of MAO-B inhibition by LG were linear and intersected the *y*-axis (Figure 3C) and secondary plots showed the K_i_ value of LG for MAO-B inhibition was 0.023 ± 0.00061 µM (Figure 3D), indicating LG is a competitive inhibitor of MAO-B.

### 2.5. Molecular Docking Simulation

**Figure 4:**
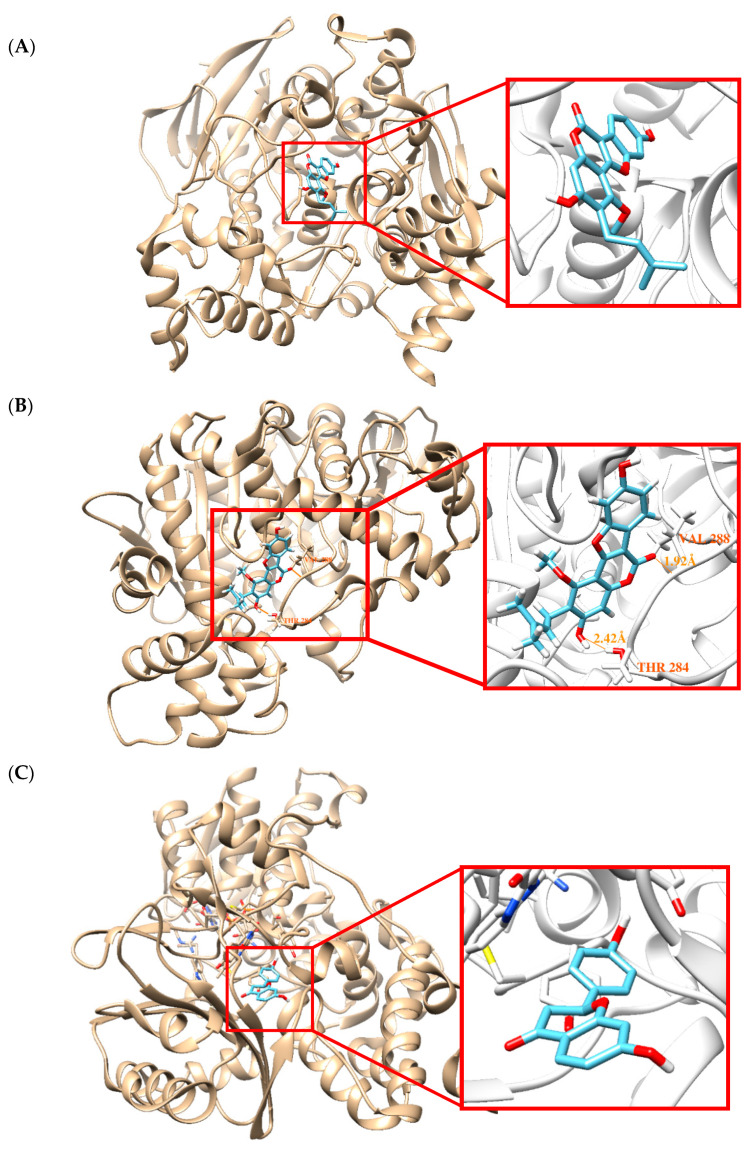

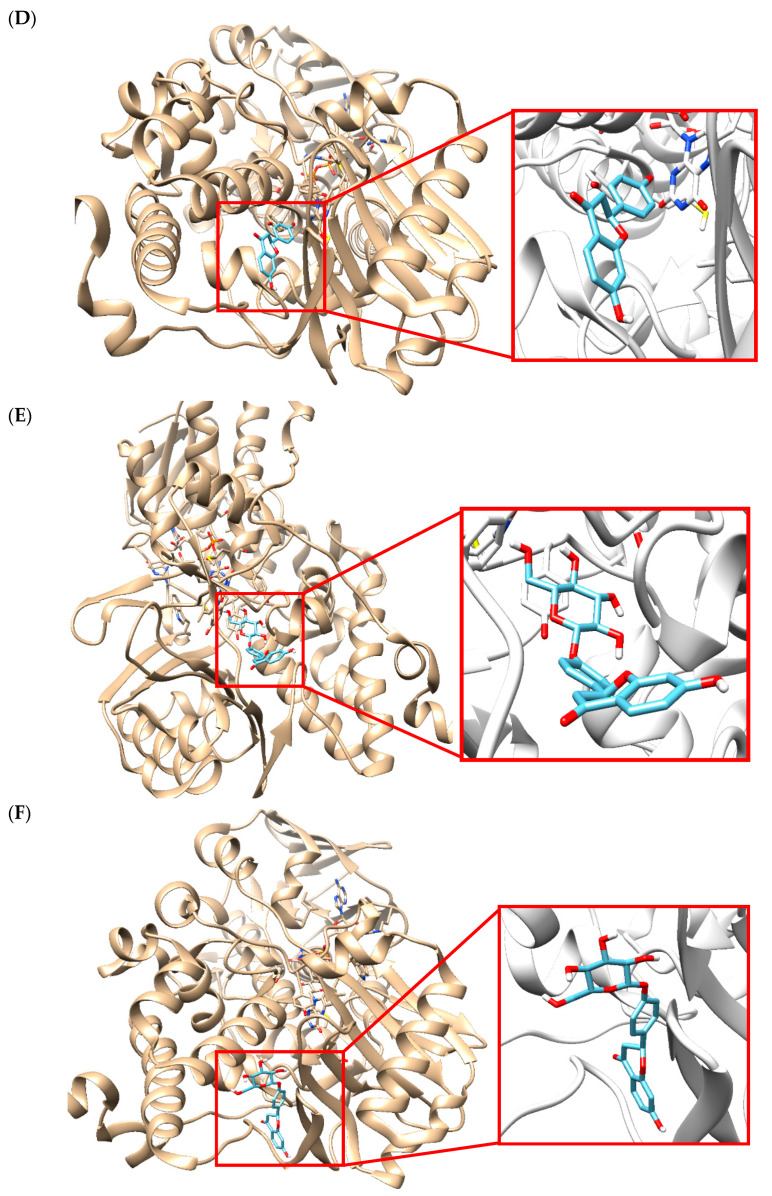
Docking simulations of GC with AChE (**A**) and BChE (**B**), LG with MAO-A (**C**) and MAO-B (**D**), and liquiritin with MAO-A (**E**) and MAO-B (**F**). AChE (1GQS), BChE (4TPK), MAO-A (2Z5X), and MAO-B (4A79) were subjected to docking analysis.

Docking simulations showed that GC located at the binding site of 3-[(1*S*)-1-(dimethylamino)ethyl]phenol (SAF) in AChE (PDB: 1GQS) and the binding site of *N*-{[(3*R*)-1-(2,3-dihydro-1H-inden-2-yl)piperidin-3-yl]methyl}-*N*-(2-methoxyethyl)naphthalene-2-carboxamide in BChE (PDB ID: 4TPK). The binding affinity (−7.8 kcal/mol) of GC for BChE was greater than its affinity for AChE (−7.1 kcal/mol) as determined by AutoDock Vina (Table 2), and these binding affinity values concurred with the IC_50_ values (Table 1). Docking simulation results suggested that GC did not form a hydrogen bond with AChE (Figure 4A), but that GC forms two hydrogen bonds with the Thr284 and Val288 residues of BChE (distances: 2.42 and 1.92 Å, respectively) (Figure 4B). These results explain the preference of GC for BChE.

LG and liquiritin located at the binding site of 7-methoxy-1-methyl-9H-beta-carboline complexed with MAO-A (PDB: 2Z5X) and of pioglitazone complexed with MAO-B (PDB: 4A79). The binding affinities of LG and liquiritin with MAO-B were greater than their binding affinities with MAO-A (Table 2), and LG binding affinities were in-line with the IC_50_ values shown in Table 1. However, docking simulations did not predict hydrogen bond formation between LG or liquiritin with MAO-A or MAO-B (Figure 4C–F).

## 3. Discussion

In the present study, GC (a coumarin) was isolated from *G. uralensis* and its BChE inhibitory activity was evaluated. Coumarins are characterized by the presence of 1,2-benzopyrone or benzopyran-2-one groups, which are the most common oxygen-containing heterocyclic compounds found in Nature. The ChE inhibitory activities by coumarins have been previously reviewed for synthetic and natural compounds [38]. Most of the known coumarins have a lower IC_50_ value for AChE than for BChE, and selectivity for AChE or BChE is dependent on scaffold substituents, as exemplified by 3-(4-aminophenyl)-coumarin derivatives [39]. Furthermore, potencies of natural coumarins for AChE or BChE are much weaker than those of synthetic analogues. Nevertheless, natural coumarins exhibit significant inhibitory activities against AChE, examples include xanthotoxin from *Ferula lutea* (IC_50_ = 0.76 μM) [40] and a 4-phenylcoumarin mesuagenin B from *Mesua elegans* (IC_50_ = 0.70 μM) [41]. Based on the classification of natural AChE inhibitors, those with IC_50_ values ≤ 15 μM are termed high potency inhibitors and those with values ranging from 15 to 50 μM moderate potency inhibitors [42]. According to this classification, GC is a high potency AChE inhibitor (IC_50_ = 14.77 μM), though the value is near the threshold. In a previous study, osthenol, a prenylated coumarin obtained from *Angelica pubescens*, selectively inhibited MAO-A, and exhibited moderate AChE inhibitory activity (IC_50_ = 25.3 μM) [43].

Natural coumarins have been reported to have low BChE inhibitory activities; sphondin and pimpinellin from *Heracleum platytaenium* inhibited BChE by 63.69% and 78.02%, respectively, at a concentration of 25 μg/mL concentration (115.7 and 101.5 μM, respectively) [44], and notably, all these IC_50_ values are higher than that of GC (IC50 = 7.22 μM) as determined in the present study.

As regards other natural compounds, inhibition of BChE by GC was greater than that by boldine (IC_50_ = 321 µM) [45], hyperforin and hyuganin C (IC_50_ = 141.60 and 38.86 μM, respectively) [46], cremaphenanthrene F (14.62) [47], scopoletin (IC_50_ = 9.11 µM) [48], and broussonin A and sagachromanol I (IC_50_ = 7. 50 and 10.79 µM, respectively) [49], but less than those of norditerpenoids isograndifoliol and (1*R*,15*R*)-1-acetoxycryptotanshinone (IC_50_ = 0.9 and 2.4 µM, respectively) [50]. Notably, these IC_50_ values were much greater than those for AChE inhibition by tannic acid (IC_50_ = 0.087 µM) [51], or hesperidin (IC_50_ = 0.00345 µM) [52].

Dual inhibitions of ChE and MAO-B have been investigated in the context of AD [11,16]. In the present study, GC potently inhibited BChE with an IC_50_ value of 7.22 μM, and moderately inhibited AChE and MAO-B, indicating GC should be considered as a multi-function inhibitor of BChE, AChE, and MAO-B.

Pan et al. reported that MAO-B inhibition by LG in rat liver mitochondria was weaker than MAO-A inhibition by a mixed type [32]. However, in our study, LG more potently inhibited human MAO-B (IC_50_ = 0.098 µM) than human MAO-A (IC_50_ = 0.27 µM) and functioned as a competitive inhibitor. The IC_50_ of LG for MAO-B was lower than that of the flavonoid acacetin (IC_50_ = 0.17 µM) [53], which is one of the lowest IC_50_ values reported for a natural compound to date. Liquiritin was less effective than LG, aglycone of liquiritin, likely observed in acacetin and acacetin 7-*O*-(6-*O*-malonylglucoside) [53].

In our docking analysis, GC showed greater binding affinity with BChE than with AChE, and LG and liquiritin were predicted to bind to MAO-B more strongly than to MAO-A, and these results agreed well with determined IC_50_ values. In particular, our kinetic study showed that GC noncompetitively inhibited BChE. Docking simulation was performed to identify BChE binding sites. The docked pose for GC indicated that it interacted with BChE beyond the active site and hydrogen bonded with Thr284 and Val288. The active-site of BChE is composed of 4 subdomains, i.e., a peripheral site, a choline binding pocket, a catalytic site, and an acyl binding pocket [54], and the acyl binding pocket contains Trp231, Leu286, and Val288, which permit binding and hydrolysis of ligands and substrates bulkier than those of AChE [54], which is considered to be largely responsible for the different ligand-binding specificities of AChE and BChE [55]. Jannat et al. reported that (2*S*,3*R*)-pretosin C is a noncompetitive inhibitor of BChE and that it hydrophobically interacts with Val288, Lue286, and Phe357, and hydrogen bonds with Gly283 and Asn397, and docks at a non-ligand binding site [56]. It was also observed that hydrogen bond formation was the main driving force behind BChE–coumarin complex formation, whereas hydrophobic and halogen interactions underpinned AChE interactions with *N*1-(coumarin-7-yl) derivatives [57]. Similarly, we found that GC hydrogen bonded with Thr284 and Val288 located outside the ligand binding site. Such results suggest that GC might bind noncompetitively at the acyl binding pocket of BChE.

## 4. Materials and Methods

### 4.1. General

The dried roots of *Glycyrrhiza uralensis* were purchased in April 2011 at a commercial herbal market (Human-herb, Gyeongsan, Gyeongbuk, South Korea). Organic solvents (e.g., methanol (MeOH), chloroform (CHCl_3_), methylene chloride (MC), ethyl acetate (EtOAc), and *n*-hexane (Hx)) were purchased from Duksan Chemical Co. (Seoul, South Korea). Column chromatography was performed using silica gel 60 (70–230 mesh, 230–400 mesh, ASTM, Merck, Darmstadt, Germany), octadecyl silica gel (ODS-A, 12 nm, S-150 m, YMC, Tokyo, Japan), and Sephadex LH-20 gel (GE Healthcare, Uppsala, Sweden). NMR spectra were recorded on a JEOL ECX-500 spectrometer, operating at 500 MHz for ^1^H- and 125 MHz for ^13^C-NMR (JEOL Ltd., Tokyo, Japan). High performance liquid chromatography (HPLC) was performed using an Agilent 1260 series system (Agilent Inc., Palo Alto, CA, USA) equipped with a binary pump, an autosampler, a column oven, a Phenomenex Kinetex C18 column (2.6 μm, 150 × 4.6 mm; Phenomenex, Torrance, CA, USA), a photodiode array detector (DAD), and an evaporative light scattering detector (ELSD). Trifluoroacetic acid (TFA, 0.1%, *v*/*v*) was used in water (solvent A) and in acetonitrile (ACN; solvent B). Gradient was applied to the elution from 95% A/5% B (0–3 min) to 0% A/100% B (3–30 min) at 0.5 mL/min using 3 μL of injection volume.

### 4.2. Extraction and Isolation of Coumarin Derivatives

The dried roots of *Glycyrrhiza uralensis* (1 kg) were extracted with MeOH at room temperature for 24 h (3 × 10 L) to obtain a crude MEOH extract, and 110 g of this extract was then suspended in 2000 mL of distilled water and partitioned versus the same volume of CHCl_3_ and EtOAc. The CHCl_3_ extract (25.2 g) obtained was separated into five fractions (GHC 1–5) by silica gel column chromatography using a Hx and EtOAc gradient (30:1 to 1:1). GHC 3 (5.2 g) was then separated by silica column chromatography using an Hx and EtOAc gradient (1:0 to 0:1) to yield ten subfractions (GHC 3-1–3-10). Subfraction GHC 3-5 (1.1 g) was subjected to reverse-phase column chromatography using ODS-A gel (50% aqueous MeOH, *v*/*v*) to obtain GC (**1**, 261.0 mg, purity: 99%). Subfraction GHC 3-7 was subjected to reverse-phase column chromatography using ODS-A gel (60% aqueous MeOH, *v*/*v*) to obtained isoliquiritin (**2**, 372.0 mg, purity: 98.3%). Pure LG (**3**, 530 mg, purity: 99%) was obtained from fraction GHC 4 using silica gel column chromatography with an Hx and EtOAc gradient (15:1 to 5:1). In addition, the EtOAc soluble extract (16.7 g) was separated into nine fractions (GHE 1–9) by silica gel column chromatography using an MC and MeOH gradient (40:1 to 4:1). Fraction GHE 2 (1.2 g) was separated by silica column chromatography using an Hx and EtOAc gradient (10:1) to obtain glycyrrhetinic acid (**4**, 113 mg, purity: 99%). Fraction GHE 6 (0.9 g) was subjected to silica gel column chromatography using an MC and MeOH gradient (15:1 to 6:1) to obtain liquiritin (**5**, 128.0 mg, purity: 99%). Fraction GHE 9 (3.6 g) was isolated by reverse-phase column chromatography using ODS-A gel (40% aqueous MeOH, *v*/*v*) to yield four fractions (GHE 9-1~9-4). Subsequently, subfraction GHE 9-3 was subjected to silica gel column chromatography using an isocratic MC-EtOAc-MeOH (3.5:3.5:1) mixture to obtain liquiritin apioside (**6**, 70.0 mg, purity: 95.1%). The water-soluble extract (20.2 g) was separated into five fractions (GHD 1–5) by chromatography on an LH-20 gel column using a H_2_O and MeOH gradient (0:1 to 1:1). Fraction GHD 4 (12.6 g) was separated by reverse-phase column chromatography using a MeOH and H_2_O gradient (5:6 to 3:2, *v*/*v*) to yield seven fractions (GHD 4-1–4-7). Subfraction GHD 4-5 was subjected to silica column chromatography using a CHCl_3_-MeOH (4:1) as eluent and yielded isoliquiritin apioside (**7**, 35.0 mg, purity: 96.3%). Glycyrrhizin was obtained from hot water extracts. The hot water extracts (28.1 g) was aggregated by reducing its pH to 2.0 with 10% H_2_SO_4_ and filtering through Whatman No. 1 paper. The precipitate obtained was suspended in distilled water (1000 mL), the pH was adjusted to 7.0 using ammonia water, and glycyrrhizin (**8**, 223.0 mg, purity: 99%) was obtained by subjecting this solution to ODS-A gel column chromatography using 60% aqueous ACN as eluant. HPLC chromatograms of the eight compounds were provided in Supplementary Figure S1.

### 4.3. Chemicals and Enzyme Assays

Enzymes (recombinant human MAO-A and MAO-B, AChE from *Electrophorus electricus*, and BChE from equine serum), substrates (kynuramine and benzylamine, acetylthiocholine iodide (ATCI), *S*-butyrylthiocholine iodide (BTCI)), inhibitors (toloxatone, lazabemide, and tacrine), and other chemicals including 5,5′-dithiobis(2-nitrobenzoic acid) (DTNB) were purchased from Sigma-Aldrich (St. Louis, MO, USA) [49,58]. The irreversible inhibitors (clorgyline and pargyline) were obtained from BioAssay Systems (Hayward, CA, USA) [59].

MAO-A and MAO-B activities were measured continuously at 316 nm for 20 min, and at 250 nm for 30 min, respectively, as described previously [60,61]. The concentrations used were; kynuramine (0.06 mM) for MAO-A and benzylamine (0.3 mM) for MAO-B. AChE activity was assayed continuously for 10 min at 412 nm using 0.2 U/mL of enzyme in the presence of 0.5 mM DTNB and 0.5 mM ATCI in 0.5 mL of reaction mixture, as previously described [49,58], based on the method developed by Ellman et al. [62]. BChE activity was assayed using the same method as AChE, except using BTCI [49]. Substrate concentrations of BTCI for BChE and benzylamine for MAO-B were 2.3- and 2.1-fold of the respective K_m_ values (0.22 and 0.14 mM).

### 4.4. Inhibitory Activities and Enzyme kinetics

Inhibitions of MAO-A, MAO-B, AChE, and BChE were initially observed at an inhibitor concentration of 10 µM. IC_50_ values of compounds exhibiting >50% inhibition were determined. Kinetic parameters, inhibition types, and K_i_ values were determined for the most potent inhibitors, i.e., GC for BChE and LG for MAO-B, as previously described [49,58]. The kinetics of BChE and MAO-B inhibitions were investigated at five different substrate concentrations; 0.05, 0.1, 0.25, 0.5 or 1.0 mM for BChE, and 0.03, 0.06, 0.15, 0.3, or 0.6 mM for MAO-B. Inhibition studies were conducted in the absence or presence of each inhibitor at about 0.5×, 1.0×, and 2.0× their IC_50_ values [58]. Inhibitory patterns and K_i_ values were determined using Lineweaver-Burk plots and secondary derivative plots.

### 4.5. Analysis of Inhibitor Reversibility

The reversibilities of BChE inhibition by GC and of MAO-B inhibition by LG were investigated by dialysis at concentrations of 2 × IC_50_ values, as previously described [63]. After preincubating GC or LG with BChE or MAO-B, respectively, for 30 min, residual activities for undialyzed and 6 h-dialyzed samples were measured; relative values for A_U_ and A_D_ were then calculated and compared with each control without inhibitor. Reversibilities were determined by comparing A_U_ and A_D_ values of inhibitors with those of references. In addition, the dilution method was used to access BChE activity recovery after inhibition by GC (i.e., after preincubating BChE with GC at 50 × IC_50_ for 15 min) and diluting to a GC concentration of 1 × IC_50_ [60]. Residual activity of the preincubated and then diluted mixture was measured and compared to those of mixtures at 1× or 50 × IC_50_ concentrations.

### 4.6. Docking Simulations of GC with AChE and BChE and of LG or Liquiritin with MAO-A and MAO-B

To simulate docking of GC with AChE or BChE, we used Autodock Vina [64], which has an automated docking facility. To define enzyme docking pockets, we used a set of predefined active sites defined using a complex of AChE with 3-[(1*S*)-1-(dimethylamino)ethyl]phenol (SAF) (PDB ID: 1GQS) or a complex of BChE with *N*-{[(3*R*)-1-(2,3-dihydro-1H-inden-2-yl)piperidin-3-yl]methyl} -*N*-(2-methoxyethyl) naphthalene-2-carboxamide (3F9) (PDB ID: 4TPK). In addition, to define MAO-A or MAO-B docking sites with LG or liquiritin, we used a set of predefined active sites obtained using MAO-A/7-methoxy-1-methyl-9*H*-β-carboline complex (PDB ID: 2Z5X) or MAO-B/pioglitazone complex (PDB ID: 4A79). To prepare for docking simulations, we performed the following steps: created 2D structures, converted 2D into 3D structures, performed energy minimization using the ChemOffice program (http://www.cambridgesoft.com) and docking simulations using Chimera [65], and checked for possible hydrogen bonding interactions using 0.4 Å and 20.0° constraints using Chimera [66].

## 5. Conclusions

GC effectively inhibited BChE and AChE (IC_50_ = 7.22 and 14.77 µM, respectively), and also moderately inhibited MAO-B (IC_50_ = 29.48 µM). LG potently inhibited MAO-B (IC_50_ = 0.098 µM) and MAO-A (IC_50_ = 0.27 µM). GC was found to be a noncompetitive inhibitor of BChE and LG to be a competitive inhibitor of MAO-B. The binding affinity of GC for BChE (−7.8 kcal/mol) was higher than its affinity for AChE (−7.1 kcal/mol), and this binding was driven by hydrogen bond formation with Thr284 and Val288 of BChE. These findings regarding the multi-inhibitory effects of GC and LG suggest that they be considered potential candidates for the treatment of Alzheimer’s disease.

## Figures and Tables

**Table 1:** Inhibitions of AChE, BChE, MAO-A, and MAO-B by compounds isolated from the roots of *Glycyrrhiza uralensis*.

Compounds	Residual Activity at 10 µM (%)				IC_50_ (µM)			
	MAO-A	MAO-B	AChE	BChE	MAO-A	MAO-B	AChE	BChE
GC	70.5 ± 1.61	74.2 ± 3.46	46.1 ± 4.40	44.6 ± 5.36	>40	29.48 ± 0.67	14.77 ± 0.19	7.22 ± 0.37
Isoliquiritin	81.8 ± 1.61	75.4 ± 2.27	69.9 ± 2.20	91.9 ± 7.16	>40	>40	>40	>40
LG	0.46 ± 1.60	0.00 ± 3.34	95.3 ± 3.59	82.5 ± 0.26	0.27 ± 0.041	0.098 ± 0.00079	>40	>40
Glycyrrhetinic acid	96.3 ± 2.64	84.0 ± 2.16	97.3 ± 1.61	95.0 ± 4.12	>40	>40	-	-
Liquiritin	93.5 ± 0.00	90.2 ± 0.56	93.5 ± 5.12	95.5 ± 4.67	>40	>40	>40	>40
Liquiritin apioside	86.9 ± 2.41	94.8 ± 0.57	63.5 ± 2.56	97.6 ± 0.93	>40	>40	36.68 ± 1.42	>40
Isoliquiritin apioside	86.6 ± 3.27	80.3 ± 5.57	93.5 ± 3.07	95.6 ± 0.88	>40	>40	-	-
Glycyrrhizin	95.8 ± 3.30	93.1 ± 4.32	97.7 ± 2.14	82.3 ± 7.95	>40	>40	-	-
Toloxatone					1.08 ± 0.025	-	-	-
Lazabemide					-	0.063 ± 0.015	-	-
Clorgyline					0.007 ± 0.00070	-	-	-
Pargyline					-	0.028 ± 0.0043	-	-
Tacrine						-	0.27 ± 0.019	0.014 ± 0.0043

-, not determined. Values above are the means ± SEs of triplicate experiments, and IC_50_ values were graphically determined at three different inhibitor concentrations around its concentration showing 50% of residual activity.

**Table 2:** Docking scores of GC, LG, and liquiritin with AChE, BChE, MAO-A, and MAO-B.

Compounds	Docking Scores (kcal/mol)			
	AChE	BChE	MAO-A	MAO-B
GC	−7.1	−7.8	-	-
LG	-	-	−7.9	−8.8
Liquiritin	-	-	−2.9	−4.1

The values were obtained using AutoDock Vina.

## Supplementary Materials

^1^H- and ^13^C-NMR spectral data are available in the Supplementary Materials.
