# Supplementary material for: Inhibition of Butyrylcholinesterase and Human Monoamine Oxidase-B by the Coumarin Glycyrol and Liquiritigenin Isolated from Glycyrrhiza uralensis
Source: Molecules. 2020 Aug 26;25(17):3896. doi: 10.3390/molecules25173896 (PMC7504216; doi:10.3390/molecules25173896)
Supplement: Supplementary file 1 [file molecules-25-03896-s001.pdf]

Supplementary Material

Glycyrol (1): White powder; ESI-MS 367 $[M+H]^+$; Molecular formula $C_{21}H_{18}O_6$; ^{1}H-NMR (500 MHz, CD_3OD) δ 7.72 (1H, d, *J*=8.4 Hz, H-7), 7.18 (1H, d, *J*=2.0 Hz, H-10), 6.97 (1H, dd, *J*=2.0, 8.4 Hz, H-8), 6.78 (1H, s, H-4), 5.20 (1H, t, H-2'), 3.91 (3H, s, OCH_3), 3.34 (2H, s, H-1'), 1.77 (3H, s, H-4'), 1.66 (3H, s, H-5'); ^{13}C-NMR (125 MHz, CD_3OD) δ 159.9 (C-3), 158.6 (C-6), 157.9 (C-11a), 156.4 (C-10a), 156.6 (C-9), 154.4 (C-1), 153.4 (C-4a), 131.4 (C-3'), 122.9 (C-2'), 120.9 (C-7), 120.2 (C-2), 114.8 (C-7a), 114.5 (C-8), 102.7 (C-6a), 100.2 (C-1a), 99.7 (C-10), 99.0 (C-4), 62.9 (OCH_3), 25.9 (C-4'), 22.5 (C-1'), 18.2 (C-5').

Isoliquiritin (2): White powder; ESI-MS 402 $[M+H]^+$; Molecular formula $C_{21}H_{22}O_9$; ^{1}H-NMR (500 MHz, CD_3OD) δ 8.01 (1H, d, *J*=9.0 Hz, H-6'), 7.84 (1H, d, *J*=15.0 Hz, H-α), 7.74 (2H, d, *J*=9.0 Hz, H-2, 6), 7.72 (1H, d, *J*=15.0 Hz, H-β), 7.18 (2H, d, *J*=9.0 Hz, H-3, 5), 6.44 (1H, dd, *J*=2.2, 9.0 Hz, H-5’), 6.32 (1H, d, *J*=2.2 Hz, H-3'), 5.01 (1H, d, *J*=7.1 Hz, H-1''), 3.72 and 3.41 (2H, m, H-6''), 3.33-3.52 (4H, m, 2'', 3'', 4'', 5''); ^{13}C-NMR (125 MHz, CD_3OD) δ 192.0 (C=O), 166.2 (C-2'), 165.2 (C-4'), 159.7 (C-4), 143.4 (C-α), 132.1 (C-6'), 130.0 (C-2, 6), 129.2 (C-1), 118.7 (C- β), 116.6 (C-3, 5), 113.3 (C-1'), 107.9 (C-5'), 102.5 (C-3'), 100.5 (C-1''), 76.9 (C-2''), 76.6 (C-3''), 73.5 (C-4''), 69.9 (C-5''), 61.1 (C-6).

Liquiritigenin (3): Colorless crystal; ESI-MS 255 $[M-H]^-$; Molecular formula $C_{15}H_{12}O_4$; ^{1}H-NMR (500 MHz, CD_3OD) δ 7.71 (1H, d, *J*=8.8 Hz, H-5), 7.31 (1H, d, *J*=8.4 Hz, H-2'), 6.82 (2H, d, *J*=8.4 Hz, H-3', 5'), 6.50 (1H, dd, *J*=8.8, 2.0 Hz, H-6), 6.35 (1H, d, *J*=2.0 Hz, H-8), 5.36 (1H, dd, *J*=13.2, 2.8 Hz, H-2), 3.05 (1H, dd, *J*=16.8, 13.2 Hz, H-3a), 2.67 (1H, dd, *J*=16.8, 2.8

Hz, H-3b); ^{13}C-NMR (125 MHz, CD_3OD) δ 193.6 (C-4), 166.9 (C-7), 165.6 (C-9), 159.0 (C-4'), 131.4 (C-1'), 129.9 (C-5), 129.1 (C-2', 6'), 116.4 (C-3', 5'), 115.0 (C-10), 111.8 (C-6), 103.9 (C-8), 81.1 (C-2), 45.0 (C-3).

Glycyrrhetinic acid (4): White powder; ESI-MS 469 $[M-H]^-$; Molecular formula $C_{30}H_{46}O_4$; ^{1}H-NMR (500 MHz, $CDCl_3$) δ 5.73 (1H, s, H-12), 3.26 (1H, dd, *J*=5.8, 10.8 Hz, H-3), 2.81 (2H, m, H-1), 2.37 (1H, s, H-9), 1.39 (3H, s, H-27), 1.25 (3H, s, H-29), 1.16 (3H, s, H-25), 1.15 (3H, s, H-26), 1.03 (3H, s, H-23), 0.86 (3H, s, H-28), 0.83 (3H, s, H-24); ^{13}C-NMR (125 MHz, $CDCl_3$) δ 200.5 (C-11), 181.8 (C-30), 169.5 (C-13), 128.5 (C-12), 78.9 (C-3), 61.8 (C-9), 54.9 (C-5), 48.2 (C-18), 45.5 (C-14), 43.8 (C-20), 43.2 (C-8), 41.0 (C-19), 39.1 (C-1, 4), 37.2 (C-10, 22), 32.8 (C-7), 31.9 (C-17), 30.9 (C-21), 28.6 (C-23, 29), 28.1 (C-28), 27.2 (C-2), 26.5 (C-15, 16), 23.4 (C-27), 18.7 (C-26), 17.5 (C-6), 16.4 (C-25), 15.6 (C-24).

Liquiritin (5): White powder; ESI-MS 417 $[M-H]^-$; Molecular formula $C_{21}H_{22}O_9$; ^{1}H-NMR (500 MHz, DMSO-d_6) δ 7.64 (1H, d, *J*=8.4 Hz, H-5), 7.44 (2H, d, *J*=8.7 Hz, H-2', 6'), 7.06 (2H, d, *J*=8.7 Hz, H-3', 5'), 6.50 (1H, dd, *J*=2.1, 8.7 Hz, H-6a), 6.34 (1H, d, *J*=2.1 Hz, H-8), 5.52 (1H, dd, *J*=2.5, 12.6 Hz, H-2), 3.46 (1H, s, H-6b), 3.07-3.40 (5H, m, H-3a, 2'', 3'', 4'', 5''), 2.66 (1H, dd, *J*=2.7, 16.7 Hz, H-3b); ^{13}C-NMR (125 MHz, DMSO-d_6) δ 190.2 (C-4), 164.9 (C-7), 163.3 (C-9), 157.7 (C-4'), 132.6 (C-5, C-1'), 128.2 (C-2', 6'), 116.4 (C-3', 5'), 113.8 (C-10), 110.8 (C-6), 102.8 (C-8), 100.5 (C-1''), 78.9 (C-2), 77.3 (C-5''), 76.8 (C-3''), 73.4 (C-2''), 69.9 (C-4''), 60.9 (C-6''), 43.4 (C-3).

Liquiritin apioside (6): Yellow powder; ESI-MS 551 $[M+H]^+$; Molecular formula $C_{26}H_{30}O_{13}$; ^{1}H-NMR (500 MHz, CD_3OD) δ 7.74 (1H, d, *J*=8.8 Hz, H-5), 7.44 (2H, d, *J*=8.6 Hz, H-2', 5'), 7.13 (2H, d, *J*=8.6 Hz, H-3', 6'), 6.52 (1H, dd, *J*=8.8, 2.0 Hz, H-6a), 6.38 (1H, d, *J*=2.0 Hz, H-

8), 5.49 (1H, br s, H-1'''), 5.45 (1H, dd, *J*=12.7, 2.7 Hz, H-2), 5.02 (1H, d, *J*=7.3 Hz, H-1''), 3.96 (1H, d, *J*=1.6 Hz, H-2'''), 3.65 (1H, dd, *J*=7.7, 9.0 Hz, H-2''), 3.04 (1H, dd, *J*=16.8, 13.0 Hz, H-3a), 2.74 (1H, d, *J*=16.8 Hz, H-3b); ^{13}C-NMR (125 MHz, CD_3OD) δ 193.6 (C-4), 167.1 (C-7), 165.7 (C-9), 159.4 (C-4'), 134.6 (C-1'), 130.1 (C-5), 129.1 (C-2', 6'), 112.1 (C-6), 117.9 (C-3', 5'), 115.2 (C-10), 111.0 (C-1'''), 104.1 (C-8), 101.0 (C-1''), 80.9 (C-2), 80.9 (C-3'''), 78.8 (C-2''), 78.3 (C-3'', C-5'', C-2'''), 75.6 (C-4'''), 71.6 (C-4''), 66.2 (C-5'''), 62.6 (C-6''), 45.1 (C-3).

Isoliquiritin apioside (7): Yellow powder; ESI-MS 549 [M-H]$^-$; Molecular formula $C_{26}H_{30}O_{13}$; ^{1}H-NMR (500 MHz, CD_3OD) δ 7.97 (1H, d, *J*=9.0 Hz, H-6'), 7.78 (1H, d, *J*=15.4 Hz, H-β), 7.72 (2H, d, *J*=8.7 Hz, H-2, 6), 7.68 (1H, d, *J*=15.4 Hz, H-α), 7.11 (2H, d, *J*=8.8 Hz, H-3, 5), 6.42 (1H, dd, *J*=9.0, 2.4 Hz, H-5'), 6.29 (1H, d, *J*=2.4 Hz, H-3'), 5.47 (1H, d, *J*=1.7 Hz, H-1'''), 5.05 (1H, d, *J*=7.3 Hz, H-1''), 4.07 (1H, d, *J*=1.7 Hz, H-5'''a), 3.96 (1H, d, *J*=1.6 Hz, H-2'''), 3.91 (1H, dd, *J*=12.3, 2.3 Hz, H-6''a), 3.80 (1H, d, *J*=9.5 Hz, H-5'''b), 3.71 (1H, m, H-6''b), 3.70 ~ 3.37 (4H, m, H2''~5''), 3.54 (2H, s, H-4'''); ^{13}C-NMR (125 MHz, CD_3OD) δ 193.7 (C=O), 167.9 (C-4'), 166.8 (C-2'), 161.2 (C-4), 145.1 (C-β), 133.7 (C-6'), 131.7 (C-2, 6), 130.7 (C-1), 120.3 (C-α), 118.1 (C-3, 5), 114.9 (C-1'), 111.1 (C-5'), 109.5 (C-1'''), 104.0 (C-3'), 100.6 (C-1''), 80.9 (C-2'''), 78.8 (C-5''), 78.7 (C-3'''), 78.3 (C-3''), 78.2 (C-2''), 75.6 (C-4'''), 71.5 (C-4''), 66.2 (C-5'''), 62.6 (C-6'').

Glycyrrhizin (8): White powder; ESI-MS 823 [M+H]$^+$; Molecular formula $C_{42}H_{62}O_{16}$; ^{1}H-NMR (500 MHz, pyridine-d_5) δ 1.06 (1H, m, H-1a), 2.05 (1H, m, H-2a), 2.29 (1H, m, H-2b), 3.04 (1H, m, H-1b), 3.35 (1H, dd, *J*=5.4, 11.6 Hz, H-3), 0.76 (1H, m, H-5), 1.51 (1H, m, H-6a), 1.29 (1H, m, H-6b), 1.58 (1H, m, H-7a), 1.25 (1H, m, H-7b), 2.45 (1H, s, H-9), 5.94 (1H, s, H-12), 1.73 (1H, m, H-22a), 1.11 (1H, m, H-15a), 2.09 (1H, m, H-21), 0.96 (1H, m, H-16a), 2.53 (1H, m, H-19), 2.14 (1H, m, H-18), 1.75 (1H, m, H-22b), 1.47 (1H, m, H-21b), 1.41 (3H,

s, H-27), 1.21 (3H, s, H-25), 1.06 (3H, s, C-28), 1.42 (3H, s, H-27), 0.81 (3H, s, H-24), 1.34 (3H, s, H-26), 5.03 (1H, d, *J*=7.9 Hz, C-1'), 4.24 (1H, dd, *J*=8.0, 9.1 Hz, C-2'), 4.36 (1H, t, *J*=8.2 Hz, C-3'), 4.52 (1H, m, C-4'), 4.54 (1H, m, C-5'), 5.37 (1H, d, *J*=7.9 Hz, C-1''), 4.21 (1H, dd, *J*=8.1, 9.6 Hz, C-2''), 4.28 (1H, m, C-3''), 4.58 (1H, m, C-4''), 4.58 (1H, t, *J*=9.4 Hz, C-5''); ^{13}C-NMR (125 MHz, pyridine-d_5) δ 199.6 (C-11), 179.2 (C-29), 172.5 (C-6'), 172.2 (C-6''), 169.6 (C-13), 128.7 (C-12), 107.0 (C-1'), 105.1 (C-1''), 89.2 (C-3), 84.5 (C-2'), 78.5 (C-2''), 77.8 (C-3'), 77.7 (C-3''), 77.3 (C-4'), 76.8 (C-4''), 73.3 (C-5''), 73.1 (C-5'), 62.1 (C-9), 55.4 (C-5), 48.7 (C-18), 45.6 (C-14), 44.1 (C-20), 43.6 (C-8), 41.8 (C-19), 40.0 (C-4), 39.6 (C-1), 38.5 (C-22), 37.2 (C-10), 33.1 (C-7), 32.2 (C-17), 31.7 (C-21), 28.8 (C-30), 28.7 (C-28), 28.2 (C-23), 26.9 (C-15), 26.7 (C-2, 16), 23.6 (C-27), 18.9 (C-26), 17.7 (C-6), 16.9 (C-25), 16.8 (C-24).

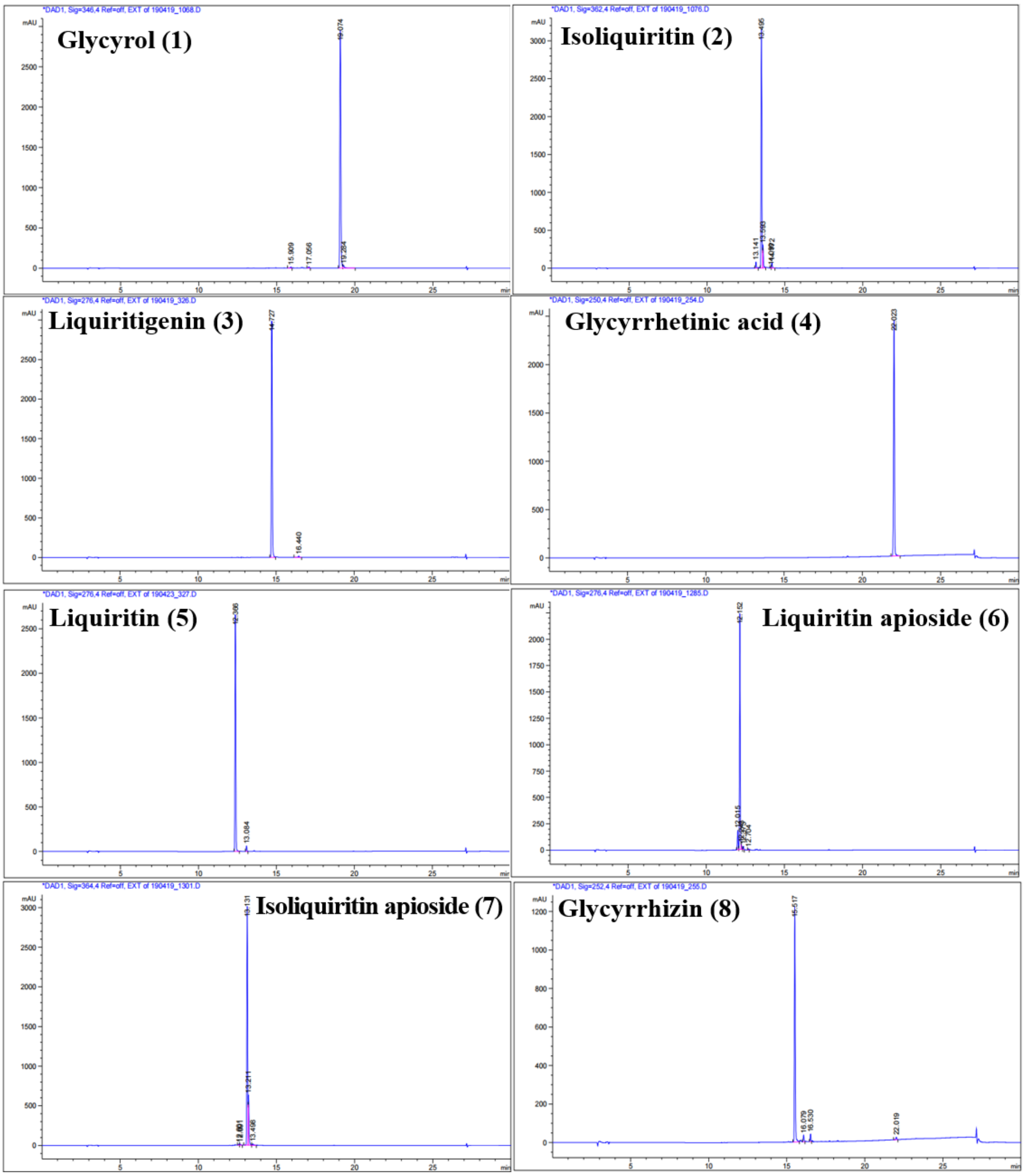

Figure S1. HPLC chromatograms of 8 compounds isolated from the roots of *Glycyrrhiza uralensis*
